# Level of information about the relationship between diabetes mellitus and periodontitis - results from a nationwide diabetes information program

**DOI:** 10.1186/2047-783X-18-6

**Published:** 2013-03-11

**Authors:** Knut Weinspach, Ingmar Staufenbiel, Sonja Memenga-Nicksch, Stefanie Ernst, Werner Geurtsen, Hüsamettin Günay

**Affiliations:** 1 Department of Conservative Dentistry, Periodontology and Preventive Dentistry, Hannover Medical School, Carl-Neuberg-Strasse 1, Hannover, 30625, Germany; 2 Institute of Biometrics, Hannover Medical School, Carl-Neuberg-Strasse 1, Hannover, 30625, Germany; 3 School of Dentistry, University of Washington, 1959 NE Pacific Street, Seattle, WA, 98195, USA

**Keywords:** Diabetes mellitus, Periodontitis, Mutual influence, Degree of knowledge, Diabetes prevention program, Oral health care program

## Abstract

**Background:**

A comprehensive knowledge about the mutual influence between diabetes and periodontitis is decisive for the successful treatment of both diseases. The present investigation aimed at assessing the diabetic and periodontal conditions and, in particular, the degree of knowledge about the relationship between diabetes and periodontitis.

**Methods:**

During a diabetes information program, 111 nondiabetics (ND), 101 type 1 diabetics (T1D), and 236 type 2 diabetics (T2D) were subject to a medical and dental examination and completed a self-administered questionnaire. Medical examination included measurements of glycated hemoglobin (HbA1c), blood glucose (BG), and body mass index (BMI). Full-mouth examination consisted of the assessment of the decayed, missing, filled teeth index (DMFT) and the periodontal screening index (PSI). Chi-square test, ANOVA, *t* test of independent samples, univariate and multivariate logistic regression models with variable selection strategies were used for statistical analyses. Due to the exploratory character of the investigation a value of *P* ≤0.05 was considered to be statistically substantial.

**Results:**

T2D had a significantly higher PSI when compared to T1D and ND (*t* test: *P* <0.001; *P* = 0.005). Approximately 90% of T2D suffered from periodontitis. In addition, diabetics with periodontitis showed a significantly higher BMI when compared to diabetics without periodontitis (multivariate logistic regression: *P* = 0.002). Almost 60% of all investigated subjects were not informed about the mutual influence between diabetes and periodontitis. T2D had almost as little information about the increased risk for periodontitis as ND.

**Conclusions:**

The data of the present investigation suggest that there is a strong association between type 2 diabetes and chronic periodontitis. The lack of awareness of the mutual influence between diabetes and periodontitis, especially in T2D, demonstrates that this topic is still neglected in dental and diabetic treatment.

## Background

Periodontitis is a common chronic disease of the tooth-supporting tissues that is caused by bacterial deposits accumulating on the tooth surface and forming a biofilm (dental plaque) [1]. Initially, these bacterial deposits induce a gingival inflammation (gingivitis) which is completely reversible. If the biofilm remains for a longer period of time, the inflammation may expand to all periodontal tissues (gingiva, periodontal ligament, root cementum, alveolar bone) and provoke irreversible attachment loss (periodontitis). According to the classification system suggested by Armitage [2], periodontitis is basically subdivided into chronic and aggressive forms. These forms essentially differ regarding the age of onset, degree of plaque accumulation, progression rate, extent of attachment loss, and the immune competence of the host. While aggressive periodontitis is characterized by an early onset of the disease, rapid progression, pronounced periodontal destruction, and a high innate susceptibility, chronic periodontitis is predominantly seen at an older age, frequently associated with high amounts of dental plaque and calculus and a greater variation in periodontal destruction [3].

The systemic effects of periodontal diseases have been of increasing interest during the past two decades. Periodontitis is an inflammatory response to a bacterial challenge and represents a portal of entry for periodontal pathogens, bacterial endotoxins, and proinflammatory cytokines [4]. Thus, the local oral inflammatory disease, periodontitis, may induce and perpetuate a systemic inflammation that may aggravate systemic diseases such as cardiovascular disease [5], pulmonary disease [6], rheumatoid arthritis [7], and diabetes mellitus [8].

Heterogeneous epidemiological data about the prevalence of periodontal diseases are available in the dental literature. Due to the advanced age of the subjects examined in the present study, the following epidemiological data predominantly focus on the prevalence of chronic periodontitis in adults and older subjects. Based on a recent report, over 47% of the adult population suffers from chronic periodontitis in the United States. For adults aged 65 years and older, 64% showed either moderate or severe periodontitis [9]. In Europe, inconsistent methods have been applied to assess the periodontal status in the past, resulting in heterogeneous epidemiological data. In a recent report, Spain, Sweden, and Switzerland ranked as the periodontally healthiest countries, while increased tooth loss and the highest prevalence of periodontal bone loss were reported in Germany: more than 53% of adult subjects (35 to 44 years) and more than 89% of older subjects (65 to 74 years) had experienced a periodontal bone loss of ≥4 mm [10].

According to the National Diabetes Fact Sheet released by the Centers for Disease Control and Prevention (CDC), 25.8 million people (= 8.3%) of the US population were affected by diabetes in 2010 [11]. These data included 18.8 million diagnosed diabetics and an estimated number of 7 million undiagnosed cases. Among US residents aged 65 years and older, 26.9% suffered from diabetes in 2010. In addition, the CDC reported that adults aged 45 years or older, who revealed poorly controlled diabetes (HbA1c >9%), were 2.9 times more likely to develop severe periodontitis when compared to nondiabetics. Thus, besides heart disease, stroke, hypertension, blindness, kidney disease, nervous system disease, and diabetic foot syndrome, periodontal diseases have to be considered as one of the major complications of diabetes.

Only incomplete epidemiological data on the prevalence of diabetes are available for Germany and most other European countries [12]. Recent data from six population-based studies estimated a prevalence of known type 2 diabetes of 8.2% in Germany [13]. In general, the incidence of type 1 and type 2 diabetes mellitus is increasing in Europe, particularly in the United Kingdom, Germany, and France [14].

For a successful treatment of periodontitis and diabetes mellitus as well, patients themselves are the most determining factor. Especially in type 2 diabetes mellitus and chronic periodontitis, specific changeable lifestyle behaviors have been identified as critical risk factors. While the development of type 2 diabetes mellitus is closely connected with the metabolic syndrome that is characterized by overweight, hypertension, dyslipidemia, and lack of physical activity [15], the etiology of chronic periodontitis is strongly associated with behavioral factors such as insufficient oral hygiene on the one hand and smoking, malnutrition, psychological stress, and excessive alcohol consumption on the other hand [16]. In this context, studies have shown that diabetes prevention programs are successful in reducing the risk for type 2 diabetes mellitus [17-19] and oral health care programs are effective in decreasing the incidence of caries and periodontitis [20-23]. Due to the mutual influence between diabetes mellitus and periodontitis, a comprehensive knowledge about this interaction seems to be decisive for a successful treatment of both diseases. Therefore, the aim of the present investigation was to evaluate the diabetic and periodontal conditions and, in particular, the state of knowledge about the relationship between diabetes mellitus and periodontitis during a nationwide diabetes information program.

## Methods

### Clinical examination and self-administered questionnaire

The present investigation was approved by the Ethical Committee of Hannover Medical School (No. 621) and performed in the context of the oral health care program of the Department of Conservative Dentistry, Periodontology and Preventive Dentistry of Hannover Medical School [24]. Some 448 subjects (244 women and 204 men) aged 59.65 ± 13.65 years were consecutively investigated for diabetes and oral health status during a nationwide diabetes information program (the so-called ‘diabetestour’, Kirchheim-Verlag, Mainz, Germany). The examination included 111 nondiabetics (ND), 101 type 1 diabetics (T1D), and 236 type 2 diabetics (T2D) who underwent a medical and dental examination and completed a self-administered questionnaire.

The medical examination during the ‘diabetestour’ was performed by a specialized staff. Glycated hemoglobin (HbA1c), blood glucose (BG), and body mass index (BMI) were determined.

The dental examination was performed by three experienced dentists using magnification glasses, portable LED lights, and disposable dental examination kits containing a sterile dental mirror and a sterile ball-tip periodontal probe (Kerr GmbH, Rastatt, Germany). Full-mouth examination consisted of the assessment of the decayed, missing, filled teeth index (DMFT) and the periodontal screening index (PSI). The DMFT is defined as the sum of teeth with carious lesions, extracted teeth, and restored teeth. Thus, high DMFT values correlate with (1) a high degree of dental treatment needs, (2) a high amount of dental restorations or (3) a combination of both. Since wisdom teeth are not included in this index, the highest possible DMFT value is 28. The PSI was introduced in 1992 by the American Academy of Periodontology (AAP) in collaboration with the American Dental Association (ADA). It provides information about the severity and treatment needs of periodontal diseases. It is subdivided into five codes. Code 0 represents healthy periodontal conditions, codes 1 and 2 indicate gingivitis, and codes 3 and 4 indicate periodontitis.

The self-administered questionnaire included questions regarding gender, age, smoking habits, oral health, history of diabetes, metabolic control, and knowledge about the relationship between diabetes mellitus and periodontitis. Knowledge about this relationship was evaluated by the questions:

1. ‘Did you know that periodontitis and diabetes negatively affect each other?’

2. ‘Did you know that diabetics are more often affected by periodontitis than nondiabetics?’

### Statistical analysis

As this study is considered to be exploratory, *P* values are assessed descriptively and considered to be of relevance for *P* ≤0.05. For the diabetes groups (T1D, T2D, ND), descriptive data concerning gender, smoking, and knowledge about the mutual influence between diabetes and periodontitis are given in cross tabs (Tables 1 and 2). Pearson´s chi square test was used to detect significant differences between the groups. Data about DMFT, PSI, HbA1c, BG, BMI, and age, were statistically analyzed using ANOVA to detect significant differences between the diabetes groups (Table 3). When significant differences were documented, a pair-wise comparison was performed using *t* tests of independent samples (Table 4).

**Table 1:** Distribution of gender and smoking habits in diabetes groups

	**Gender**		**Smoking**		**Total**
	**Female**	**Male**	**Yes**	**No**	
**ND**	71	40	5	106	111
**T1D**	64	37	19	82	101
**T2D**	109	127	12	224	236
**Total**	244	204	36	412	448

Pearson´s chi square test: gender: *P* = 0.001; smoking habit: *P* <0,001. *ND* nondiabetic, *T1D* type 1 diabetic, *T2D* type 2 diabetic.

**Table 2:** Degree of knowledge about the correlation between diabetes mellitus and periodontitis

	**Did you know that periodontitis and diabetes negatively affect each other?**		**Did you know that diabetics are more often affected by periodontitis than nondiabetics?**		**Total**
	**Yes**	**No**	**Yes**	**No**	
**ND**	42 (37.8%)	69 (62.2%)	37 (33.3%)	74 (66.7%)	111
**T1D**	65 (64.4%)	36 (35.6%)	64 (63.4%)	37 (36.6%)	101
**T2D**	90 (38.1%)	146 (61.9%)	79 (33.5%)	157 (66.5%)	236
**Total**	197 (44.0%)	251 (56.0%)	180 (40.2%)	268 (59.8%)	448

Pearson´s chi-square test: *P* <0.001. *ND* nondiabetic, *T1D* type 1 diabetic, *T2D* type 2 diabetic.

**Table 3:** Statistically significant differences of dental and medical parameters between diabetes groups

	**ND**		**T1D**		**T2D**		***P *value**
	**N**	**MV ± SD**	**N**	**MV ± SD**	**N**	**MV ± SD**	
**DMFT**	111	17.81 ± 6.36	101	16.12 ± 7.58	236	18.80 ± 5.47	0.001
**PSI**	111	3.26 ± 0.89	100	2.79 ± 1.29	234	3.52 ± 0.74	<0.001
**HbA1c**	36	6.15 ± 1.26	99	7.21 ± 0.98	210	6.84 ± 0.80	<0.001
**BG**	110	105.70 ± 24.18	99	142.18 ± 51.69	236	142.03 ± 46.87	<0.001
**BMI**	107	26.59 ± 6.75	98	25.65 ± 5.25	228	29.05 ± 6.08	<0.001
**Age**	110	60.41 ± 12.77	101	48.99 ± 16.21	231	63.95 ± 9.85	<0.001

One-way ANOVA; *ND* nondiabetic, *T1D* type 1 diabetic, *T2D* type 2 diabetic; *MV* mean value; *SD* standard deviation, *DMFT* decayed, missing, filled teeth index, *PSI* periodontal screening index; *HbA1c*, glycated hemoglobin; *BG* blood glucose; *BMI* body mass index.

**Table 4:** Pair-wise comparison of dental and medical parameters between diabetes groups

	**ND vs. T1D**	**ND vs. T2D**	**T1D vs. T2D**
**DMFT**	0.079	0.137	<0.001
**PSI**	0.002	0.005	<0.001
**HbA1c**	<0.001	<0.001	<0.001
**BG**	<0.001	<0.001	0.979
**BMI**	0.272	0.001	<0.001
**Age**	<0.001	0.005	<0.001

*t* test of independent samples; *ND* nondiabetic, *T1D* type 1 diabetic, *T2D* type 2 diabetic, *DMFT* decayed missing filled teeth index, *PSI* periodontal screening index, *HbA1c* glycated hemoglobin, *BG* blood glucose, *BMI* body mass index.

Univariate logistic regression analyses for dependent factors HbA1c (≥8%, <8%) and PSI (≥3, <3) were performed. The analyses contained the following independent parameters: age (≥65 years, <65 years), gender (male, female), smoking (yes, no), question: ‘Did you know that periodontitis and diabetes negatively affect each other?’ (yes, no), question: ‘Did you know that diabetics are more often affected by periodontitis than nondiabetics?’ (yes, no), blood glucose (mg/dl), body mass index (BMI), and the converse dependent factor (HbA1c or PSI). To estimate the variable influence, relevant parameters with *P* <0.3 were used in a multivariate logistic regression analysis with backward selection and Akaike Information Criterion (AIC) as stop criterion. Two-sided 95% confidence intervals for the odds ratios derived from the best selected logistic regression model, as mentioned before, were shown. If the lower margin is higher than 1 it may be concluded that, in the presence of a variable level, the risk of a worse outcome of PSI and HbA1c respectively, is relevant (Tables 5, 6, 7). IBM SPSS Statistics 19.0 (IBM Corporation, Somers, NY, USA) and SAS 9.2 (SAS Institute Inc., Cary, NC, USA) were used for statistical analyses.

**Table 5:** Results of multivariate logistic regression analysis of the peridontal screening index (PSI) (1/3)

**Qualitative variables**		**PSI <3**	**PSI ≥3**	***P *value**	**OR [CI]**
**Age**	**<65 years**	41	148	0.020	2.395 [1.1; 5.0]
	**≥65 years**	16	129		
**Gender**	**Female**	41	132	<0.001	3.622 [1.8; 7.5]
	**Male**	16	145		

*OR* odds ratio, *CI* confidence interval.

**Table 6:** Results of multivariate logistic regression analysis of the peridontal screening index (PSI) (2/3)

**Quantitative variables**	**PSI**	**N**	**MV ± SD**	***P *value**	**OR [CI]**
**BMI**	**Edentulous**	3	24.400 ± 4.503	0.0024	1.097 [1.0; 1.2]
	**<3**	53	26.270 ± 6.243		
	**≥3**	270	28.418 ± 5.960		

*MV* mean value, *SD* standard deviation, *OR* odds ratio, *CI* confidence interval; *BMI* body mass index.

**Table 7:** Results of multivariate logistic regression analysis of glycated hemoglobin (Hba1c) (3/3)

**Qualitative variables**		**HbA1c <8**	**HbA1c ≥8**	***P *value**	**OR [CI]**
**Smoking**	**No**	249	29	0.002	4.384 [1.7; 11.2]
	**Yes**	21	10		

*OR* odds ratio; *CI* confidence interval.

## Results

The analysis of the descriptive data revealed that the majority of ND and T1D were women whereas the group of T2D comprised more men. Approximately one in twenty subjects was smoking in the ND and T2D group, while one in five subjects was smoking in the T1D group.

Analysis of variance (ANOVA) revealed statistically significant differences between the diabetes groups for all investigated parameters.

Lowest DMFT was measured for T1D (16.12 ± 7.58). T2D (18.80 ± 5.47) had a significantly higher DMFT when compared to T1D (*t* test: *P* <0.001).

Lowest PSI was measured for T1D (2.79 ± 1.29). ND (3.26 ± 0.89) and T2D (3.52 ± 0.74) had a significantly higher PSI when compared to T1D (*t* test: *P* = 0.002; *P* <0.001). T2D revealed a significantly higher PSI when compared to ND (*t* test: *P* = 0.005). Summation of PSI code 3 and 4 revealed that approximately 85% of ND, 65% of T1D and 90% of T2D were affected by periodontitis (Table 8).

**Table 8:** Peridontal screening index (PSI) code distribution in diabetes groups

**PSI**	**ND**		**T1D**		**T2D**	
	**N**	**%**	**N**	**%**	**N**	**%**
**0**	3	2.7	10	9.9	2	0.8
**1**	1	0.9	5	5.0	1	0.4
**2**	12	10.8	20	19.8	19	8.1
**3**	43	38.7	26	25.7	63	26.7
**4**	52	46.8	39	38.6	149	63.1
**Edentulous**			1	1.0	2	0.8

*ND* nondiabetic, *T1D* type 1 diabetic, *T2D* type 2 diabetic.

Highest HbA1c was detected in T1D (7.21 ± 0.98). ND (6.15 ± 1.26) had a significantly lower HbA1c when compared to T1D and T2D (6.84 ± 0.80) (*t* test: *P* <0.001; *P* <0.001). T1D revealed a significantly higher HbA1c when compared to T2D (*t* test: *P* <0.001).

Lowest BG was recorded for ND (105.70 ± 24.18). T1D (142.18 ± 51.69) and T2D (142.03 ± 46.87) had a significantly higher BG when compared to ND (*t* test: each *P* <0.001).

Highest mean BMI was identified for T2D (29.05 ± 6.08). T2D showed a significantly higher BMI when compared to ND (26.59 ± 6.75; *P* = 0.001) and T1D (25.65 ± 5.25; *P* <0.001).

For PSI as a dependent variable, multivariate logistic regression analysis revealed that older diabetics (≥65 years) were 2.4 times more likely to develop periodontitis when compared to younger diabetics (<65 years). Male diabetics were 3.6 times more likely to suffer from a periodontal disease when compared to female diabetics. In addition, diabetics with periodontitis had a significantly higher BMI when compared to diabetics without periodontitis (*P* = 0.0024).

For HbA1c as a dependent variable, multivariate logistic regression analysis indicated that smoking diabetics were 4.4 times more likely to have elevated HbA1c levels (≥8) when compared to nonsmoking diabetics.

Responding to the question ‘Did you know that periodontitis and diabetes negatively affect each other?’ T1D significantly more often answered ‘yes’ when compared to ND and T2D (Pearson´s chi-square-test: *P* <0.001). For the question ‘Did you know that diabetics are more often affected by periodontitis than nondiabetics?’ a comparable result was detected (Pearson´s chi-square test: *P* <0.001). Altogether more than 55% of all investigated subjects were not informed about the mutual influence between periodontitis and diabetes. With regard to diabetics, T1D were significantly better informed about the correlation when compared to T2D. The sources of information, including family doctors, experts on diabetes, dentists, the media, and others are given in Table 9. In both diabetes groups the most frequently named sources were dentists (T1D: 25.7%; T2D: 16.5%) and the media (T1D: 25.7%; T2D: 12.7%).

**Table 9:** Source of information about the correlation between diabetes mellitus and periodontitis

	**ND**		**T1D**		**T2D**	
	**N**	**%**	**N**	**%**	**N**	**%**
**Family doctor**	4	3.6	3	3.0	21	8.9
**Diabetologist**	2	1.8	18	17.8	23	9.7
**Dentist**	7	6.3	26	25.7	39	16.5
**Media**	19	17.1	26	25.7	30	12.7
**Others**	9	8.1	12	11.9	13	5.5
**Total**	41	36.9	85	84.1	126	53.3

*ND* nondiabetic; *T1D* type 1 diabetic; *T2D* type 2 diabetic.

## Discussion

Both periodontitis and diabetes mellitus are frequent chronic diseases and generate enormous costs for the public health care system. Numerous studies [25,26], review articles [27,28], and meta-analyses [29,30] indicated a mutual influence between periodontitis and diabetes mellitus.

The mechanisms, whereby diabetes may negatively influence periodontal health, are primarily based on the impaired local immune defense and a reduced renewal of the periodontal tissues. In diabetic subjects, the altered functions of the immune cells result in a reduced elimination of periodonto-pathogenic bacteria on the one hand and an increased secretion of proinflammatory cytokines on the other hand [27]. These proinflammatory cytokines promote periodontal destruction and thus play a decisive role in the pathogenesis of periodontitis. Moreover, higher levels of advanced glycation end products (AGE) can be found in the periodontium of diabetics compared to nondiabetic subjects [31]. The interaction between AGEs and collagen generates highly stable collagen macromolecules, that are resistant to physiologic enzymatic degradation [32]. Hence, the renewal of all periodontal tissues is effectively compromised in diabetic subjects, especially when glycemic control is poor. These phenomena explain in part why diabetic patients are three times more likely to develop periodontitis than nondiabetic subjects. Conversely, periodontitis may negatively influence diabetes by contributing to insulin resistance thereby aggravating glycemic control. In subjects with periodontitis, even the simple act of chewing can cause a systemic dissemination of periodontal pathogens and their metabolic products [33]. This dissemination may induce a bacteremia or endotoxemia that is characterized by increased serum levels of inflammatory mediators such as C-reactive protein (CRP), IL-6, and fibrinogen. In this context, the scientific data have demonstrated that periodontal therapy may reduce the amount of circulating proinflammatory mediators and thus may contribute to the amelioration of the glycemic status [34-37]. It may be concluded from these findings that dentists, diabetologists, as well as diabetic patients should be aware of the relationship between periodontitis and diabetes.

Until now, only scarce data existed on the level of information regarding the relationship between periodontitis and diabetes mellitus. Therefore, the aim of the present study was to evaluate the dental and periodontal health in T1D and T2D and, in particular, their state of knowledge concerning the relationship between periodontitis and diabetes. However, it has to be considered that the subjects of this study participated voluntarily in a diabetes information program. Therefore, it may be concluded that our study population consisted of higher-than-average interested diabetic patients and their family members.

Our data show that T2D exhibited a significantly higher DMFT compared to T1D and that both ND and T2D demonstrated a significantly higher PSI compared to T1D. These findings were predominantly caused by the fact that T1D were significantly younger than ND and T2D. This hypothesis is supported by the fact that the older diabetics (≥65 years) were 2.4 times more likely to be affected by periodontitis when compared to younger diabetics (<65 years).

A closer look at the PSI code distribution indicates that the majority of T2D (63.1%) suffered from severe periodontitis (PSI = 4), whereas only 26.7% had a moderate periodontitis (PSI = 3). With regard to T1D, 38.6% suffered from severe periodontitis and 25.7% from moderate periodontitis. In comparison with the older study population (65- to 74-year-old subjects) of the Fourth German Dental Health Survey (DMS IV) [38], T2D demonstrated a higher and T1D a comparable fraction of subjects with severe periodontitis. Since both diabetes groups (T1D and T2D) were on average younger than the older study population of the DMS IV, it can be concluded that diabetes may enhance periodontal destruction and may act as a predisposing factor for periodontitis. The negative influence of diabetes on periodontal health, which was the subject of clinical trials [39,40], review articles [27,28], and meta-analyses [29,41], is now generally accepted.

The whole study population was, on average, overweight. However, T2D had a significantly higher BMI compared to ND and T1D. The subjects of this group were close to the threshold of obesity. In addition, diabetics with periodontitis (PSI ≥3) demonstrated a significantly higher BMI when compared to diabetics without periodontitis (PSI <3). These findings document that overweight and obesity may negatively influence periodontal integrity and thus may result in periodontal destruction. Interestingly, Suvan *et al*. [42] recently reported that with an odds ratio of 2.13, overweight or obese individuals are more susceptible to periodontitis when compared to individuals with a normal BMI. It was concluded that obesity may cause a subclinical inflammatory response that is characterized by increased levels of acute-phase proteins, proinflammatory cytokines and leukocytes. Particularly proinflammatory cytokines released by the adipose tissues play a decisive role in the pathogenesis of periodontitis [43].

In addition, multivariate logistic regression analysis indicated a strong correlation between smoking and elevated HbA1c values. Similar results have been published by Nilsson *et al*. [44] who reported that smoking was associated with poor glycemic control independently of confounding factors like BMI, age, diabetes duration, and so on. According to Nilsson *et al*. this association may be (1) a direct result of tobacco consumption, which increases insulin resistance and promotes the insulin resistance syndrome, or (2) a consequence of an adverse, smoking-related lifestyle, which is associated with a reduced compliance regarding anti-diabetic treatment.

The results of the present study indicate insufficient oral health awareness in diabetic patients, and particularly in T2D. A total of 56% of the participants of this study had an insufficient knowledge about the mutual influence between diabetes and periodontitis. T2D had a significantly lower level of information when compared to T1D. Thus, 62% of T2D did not know that periodontitis and diabetes may negatively influence each other. In addition, 66% did not know that patients with diabetes are more often affected by periodontitis than nondiabetic individuals. These data reveal that T2D have almost as little information about the increased risk for periodontitis as ND. The different level of information in T1D and T2D may be caused by the differences in age of onset of the disease, pathophysiology, clinical symptoms, and treatment modalities. Patients of group T1D are confronted with their disease early in life and therefore depend on a continuous lifelong insulin therapy. Diabetic patients of group T2D however, usually develop hyperglycemia latently and do not always require daily medication. In addition, most T2D are obese or have an increased percentage of body fat predominantly in the abdominal region [45]. It may be concluded that patients of group T1D are more compliant and have a more profound interest in their disease when compared to T2D.

Our findings about the deficiency of oral health information in diabetics are similar to other clinical trials. For instance, Bowyer *et al*. [46] reported, that 69.1% of the interviewed study participants were never advised by health care professionals on the correlation of oral hygiene with diabetes. A Jordanian study indicated that only 47.7% of the investigated diabetic patients were aware that they are more often affected by gum diseases and oral health complications than nondiabetic individuals [47].

## Conclusion

The results of the present investigation indicate a strong correlation between type 2 diabetes and (1) periodontitis and (2) a deficient knowledge about the mutual influence between diabetes and periodontitis. This lack of knowledge emphasizes the need for more information about oral health care in patients with diabetes mellitus. In particular, dentists and diabetologists should inform diabetic patients about their increased risk for periodontitis and the importance of an excellent compliance in diabetic and periodontal treatment as well.

### A comprehensive oral health care strategy for dental practitioners should include

1. Oral examination (detection of caries, periodontitis, and mucosal diseases)

2. Information about

a. The pathogenesis of caries and periodontitis

a. The systemic effect of gingival and periodontal inflammations

a. A balanced and ‘tooth-friendly’ diet

3. Treatment of the entire oral cavity (whole-mouth therapy concept).

## Competing interests

The authors declare that they have no competing interests.

## Authors’ contributions

KW participated in the conception of the study, the acquisition, analysis and interpretation of data, and drafted the manuscript. IS participated in the acquisition of data and critically revised the manuscript. SN participated in the acquisition of data. SE substantially contributed to the analysis of data. WG participated in drafting the manuscript. HG participated in the conception of the study, the acquisition, analysis and interpretation of data, and critically revised the manuscript. All authors read and approved the final manuscript.
